# Hydrogel-Forming Microneedle Arrays Allow Detection of Drugs and Glucose *In Vivo*: Potential for Use in Diagnosis and Therapeutic Drug Monitoring

**DOI:** 10.1371/journal.pone.0145644

**Published:** 2015-12-30

**Authors:** Ester Caffarel-Salvador, Aaron J. Brady, Eyman Eltayib, Teng Meng, Ana Alonso-Vicente, Patricia Gonzalez-Vazquez, Barbara M. Torrisi, Eva Maria Vicente-Perez, Karen Mooney, David S. Jones, Steven E. J. Bell, Colin P. McCoy, Helen O. McCarthy, James C. McElnay, Ryan F. Donnelly

**Affiliations:** 1 School of Pharmacy, Queen’s University Belfast, 97 Lisburn Road, Belfast, BT97BL, United Kingdom; 2 School of Chemistry and Chemical Engineering, Queen’s University Belfast, Stranmillis Road, Belfast, BT9 5AG, United Kingdom; Michigan State University, UNITED STATES

## Abstract

We describe, for the first time the use of hydrogel-forming microneedle (MN) arrays for minimally-invasive extraction and quantification of drug substances and glucose from skin *in vitro* and *in vivo*. MN prepared from aqueous blends of hydrolysed poly(methyl-vinylether-co-maleic anhydride) (11.1% w/w) and poly(ethyleneglycol) 10,000 daltons (5.6% w/w) and crosslinked by esterification swelled upon skin insertion by uptake of fluid. Post-removal, theophylline and caffeine were extracted from MN and determined using HPLC, with glucose quantified using a proprietary kit. *In vitro* studies using excised neonatal porcine skin bathed on the underside by physiologically-relevant analyte concentrations showed rapid (5 min) analyte uptake. For example, mean concentrations of 0.16 μg/mL and 0.85 μg/mL, respectively, were detected for the lowest (5 μg/mL) and highest (35 μg/mL) Franz cell concentrations of theophylline after 5 min insertion. A mean concentration of 0.10 μg/mL was obtained by extraction of MN inserted for 5 min into skin bathed with 5 μg/mL caffeine, while the mean concentration obtained by extraction of MN inserted into skin bathed with 15 μg/mL caffeine was 0.33 μg/mL. The mean detected glucose concentration after 5 min insertion into skin bathed with 4 mmol/L was 19.46 nmol/L. The highest theophylline concentration detected following extraction from a hydrogel-forming MN inserted for 1 h into the skin of a rat dosed orally with 10 mg/kg was of 0.363 μg/mL, whilst a maximum concentration of 0.063 μg/mL was detected following extraction from a MN inserted for 1 h into the skin of a rat dosed with 5 mg/kg theophylline. In human volunteers, the highest mean concentration of caffeine detected using MN was 91.31 μg/mL over the period from 1 to 2 h post-consumption of 100 mg Proplus^®^ tablets. The highest mean blood glucose level was 7.89 nmol/L detected 1 h following ingestion of 75 g of glucose, while the highest mean glucose concentration extracted from MN was 4.29 nmol/L, detected after 3 hours skin insertion in human volunteers. Whilst not directly correlated, concentrations extracted from MN were clearly indicative of trends in blood in both rats and human volunteers. This work strongly illustrates the potential of hydrogel-forming MN in minimally-invasive patient monitoring and diagnosis. Further studies are now ongoing to reduce clinical insertion times and develop mathematical algorithms enabling determination of blood levels directly from MN measurements.

## Introduction

Therapeutic drug monitoring (TDM) aims to promote optimum clinical treatment by maintaining drug levels within a defined therapeutic range [1]. Typically, TDM is limited to drugs with a narrow therapeutic window and is most commonly used to detect and prevent toxicity and sub-therapeutic dosing in vulnerable patient populations, such as the elderly, neonates and those with organ dysfunction [2]. However, TDM also plays a crucial role in maintaining health in many diverse populations. It is regularly performed in all patients who have received any form of organ transplantation, is a mainstay of control in many forms of epilepsy and is personally performed by the majority of diabetic patients on a daily basis. Unfortunately, given the predisposition of neonates to infection, they frequently require potent antimicrobial therapy with drugs such as amikacin, gentamicin and vancomycin, all with narrow therapeutic windows and potential for toxicity. Similarly, in adults receiving potent antimicrobial agents, such as aminoglycosides and glycopeptides, TDM is regularly recommended. As liver and renal function in the elderly, neonatal and critically unwell populations can be significantly different from that of older infants and adults, the clearance of these potentially-toxic drugs is, therefore, a significant issue.

In addition to infection, the most common metabolic problem in neonates, neonatal hypoglycaemia, is frequently associated with serious complications, such as impaired neurological development. Although blood glucose monitoring (BGM) is regularly performed without issue in many adult patient groups, problems associated with BGM in neonates are compounded by an already limited neonatal blood volume. Therefore, neonatal BGM, typically performed four times daily, is a significant contributory factor to iatrogenic anaemia. To date, the majority of neonatal monitoring has entailed direct blood (*via* heel-prick or venous) sampling. This process is associated with undesirable effects, such as pain, bruising, scarring, iatrogenic anaemia, psychological aversion and risk of infection due to hypodermic needle use or heel-prick skin penetration. Therefore, in neonates and a large proportion of other patient cohorts, a minimally-invasive method of drug and clinical biomarker detection and monitoring would be a highly desirable therapeutic tool, negating direct blood sampling.

Using neonates as an example, interstitial fluid (ISF) at birth has a volume proportionally three-times greater than in healthy adults, suggesting a promising reservoir for drug and biomarker monitoring. Indeed, ISF concentrations often accurately reflect free (unbound and hence pharmacologically-active) concentrations of drugs and biomarkers in human plasma. In fact, tissue concentrations are usually more predictive of clinical outcome than total (i.e. free + bound) plasma concentrations [3, 4]. However, as the *stratum corneum*, the outermost layer of the skin, has evolved into an efficient barrier to outward migration of body fluids, a suitable technique is required to extract sufficient quantities of ISF for analysis. Reverse iontophoresis (RI) and clinical microdialysis (CM) have previously been proposed as means to utilise ISF in TDM. However, in RI, bulky, complex and expensive apparatus, requiring specialist operation, is often required. In addition, anions cannot be extracted in appreciable quantities, due to the net negative charge of skin, while lag times of several hours and patient perspiration can delay and compromise accuracy^3^. Similarly, in CM, the probe is often difficult to position and must be done by suitably-trained medical personnel. Furthermore, tissue trauma at the site of probe insertion frequently impairs measurement [4].

Microneedle arrays (MN) are minimally-invasive devices (50–900 μm in height, up to 2000 MN cm^−2^) that by-pass the skin’s *stratum corneum* barrier without causing any pain or bleeding [5–7]. Although MN technology has been extensively investigated in recent years, research has typically focussed on their use in transdermal delivery of drugs and intradermal vaccine administration. However, several recent studies have reported MN technology may also facilitate monitoring of physiologically relevant substances, such as glucose, glutamate and lactic acid [8–10]. Previous studies using solid silicon MN have shown the potential for biomarker capture in animal models, demonstrating how MN coated with capture proteins can bind to infection biomarkers and antibodies [11–14]. Crucially, this ability of MN to enable clinically relevant biomarker detection, potentially in real time, would provide invaluable diagnostic information, factors often compromised in the developing world. However, to date, no study has shown MN-mediated biomarker capture in human subjects.

**Fig 1:**
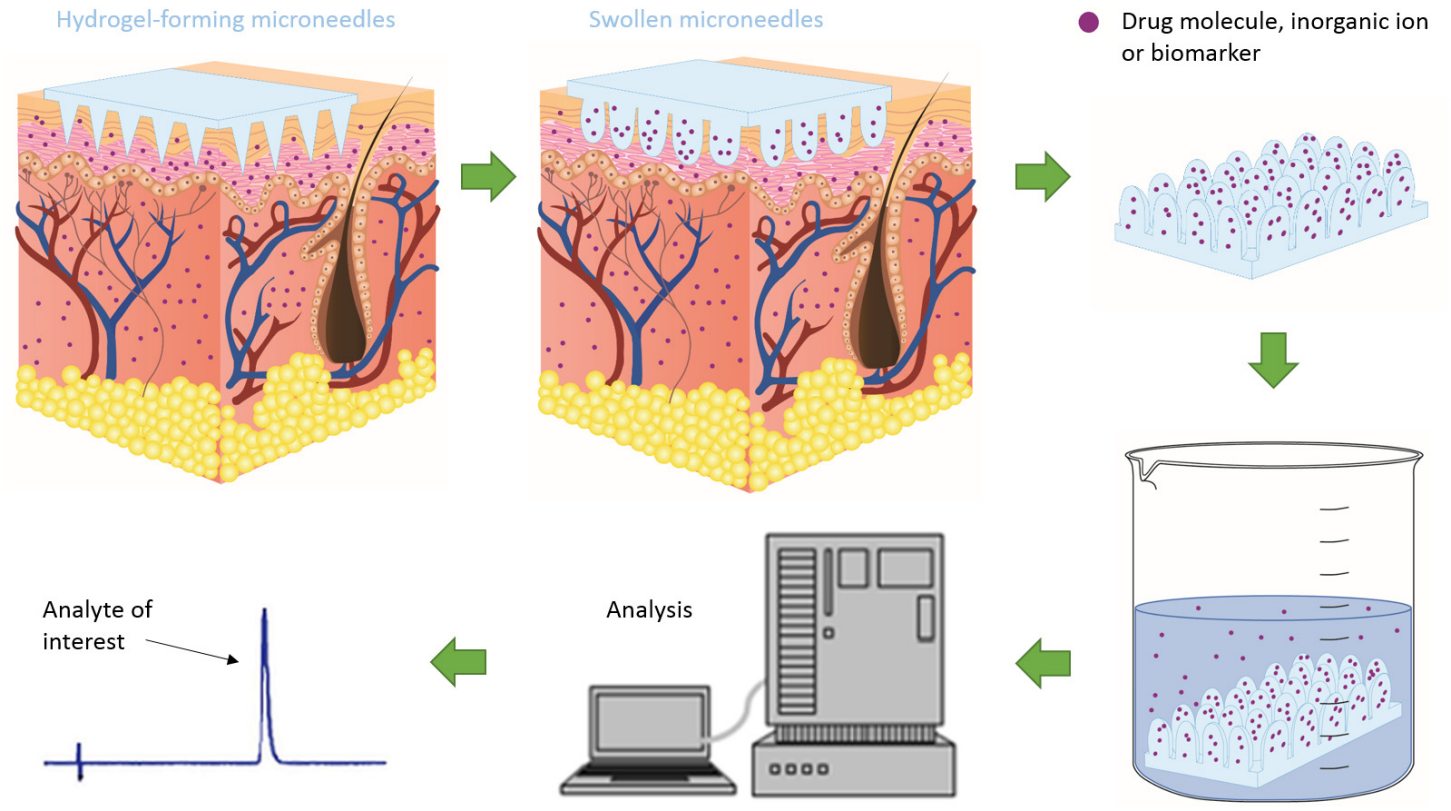
Schematic representation demonstrating the principle of MN-mediated TDM. Images display swelling of hydrogel MN upon insertion due to ISF uptake, capture and extraction of analyte of interest and ultimately quantification of analyte.

Unique hydrogel-forming MN arrays prepared from crosslinked poly(methylvinylether-*co*-maleic acid), as previously described by our Group, bypass the *stratum corneum* and swell rapidly by uptake of skin ISF [15] (Fig 1). Studies in human volunteers have shown our hydrogel-forming MN to be biocompatible and non-irritant, thus raising no safety concerns [15]. We first proposed the use of these MN for capture of skin interstitial fluid in our 2007 [16] patent and exemplified this concept in projects funded by the UK’s Engineering and Physical Sciences Research Council [17] and Action Medical Research [18]. Recently, Prausnitz *et al*. have used MN prepared from these materials to extract marker substances from skin *in vitro* and in an animal model [19]. Here we describe, for the first time, use of our hydrogel-forming MN in extraction and quantification of clinically-relevant substances theophylline, caffeine and glucose from animals and human volunteers *in vivo*.

## Materials and Methods

All research involving human participants was approved by QUB School of Pharmacy research Ethics Committee and all clinical investigation were conducted according to the principles expressed in the Declaration of Helsinki. Informed written consent, was obtained from all participants. All animal experiments throughout this study were approved by the Ethics Committee of the QUB Biological resource Unit and conducted according to the policy of the Federation of European Laboratory Animal Science Associations (FELASA) and The European Convention for the protection of vertebrate Animals used for Experimental and Other Scientific Purposes, with implementation of the principle of the 3Rs (replacement, reduction, refinement). Anaesthesia was not required, since all procedures used were mild. Any animals with 20% weight loss during the study were to be removed for euthanasia, but this did not occur. No skin reactions to microneedles occurred either. At the end of the experiment, euthanasia was by carbon dioxide

### Chemicals

Poly(methylvinylether-*co*-maelic anhydride) (Gantrez^®^ AN-139) was a gift from Ashland, (Kidderminster, UK). Poly(ethyleneglycol) 10,000 daltons and the drug substances caffeine, theophylline and 7β– hydroxyethyltheophylline (7-BHT) were obtained from Sigma-Aldrich, Poole, Dorset, UK. Glucose for *in vitro* and *in vivo* studies was obtained from VWR^®^ (Geldenaaksebaan 464, 3001, Belgium) and Thornton & Ross Ltd. (Huddersfield, HD7 5QH, England), respectively. Isoflurane (Isoflo^®^) was obtained from Abbott Laboratories, Illinois, USA. All other chemicals were of analytical reagent grade.

### Preparation of hydrogel-forming MN

**Fig 2:**
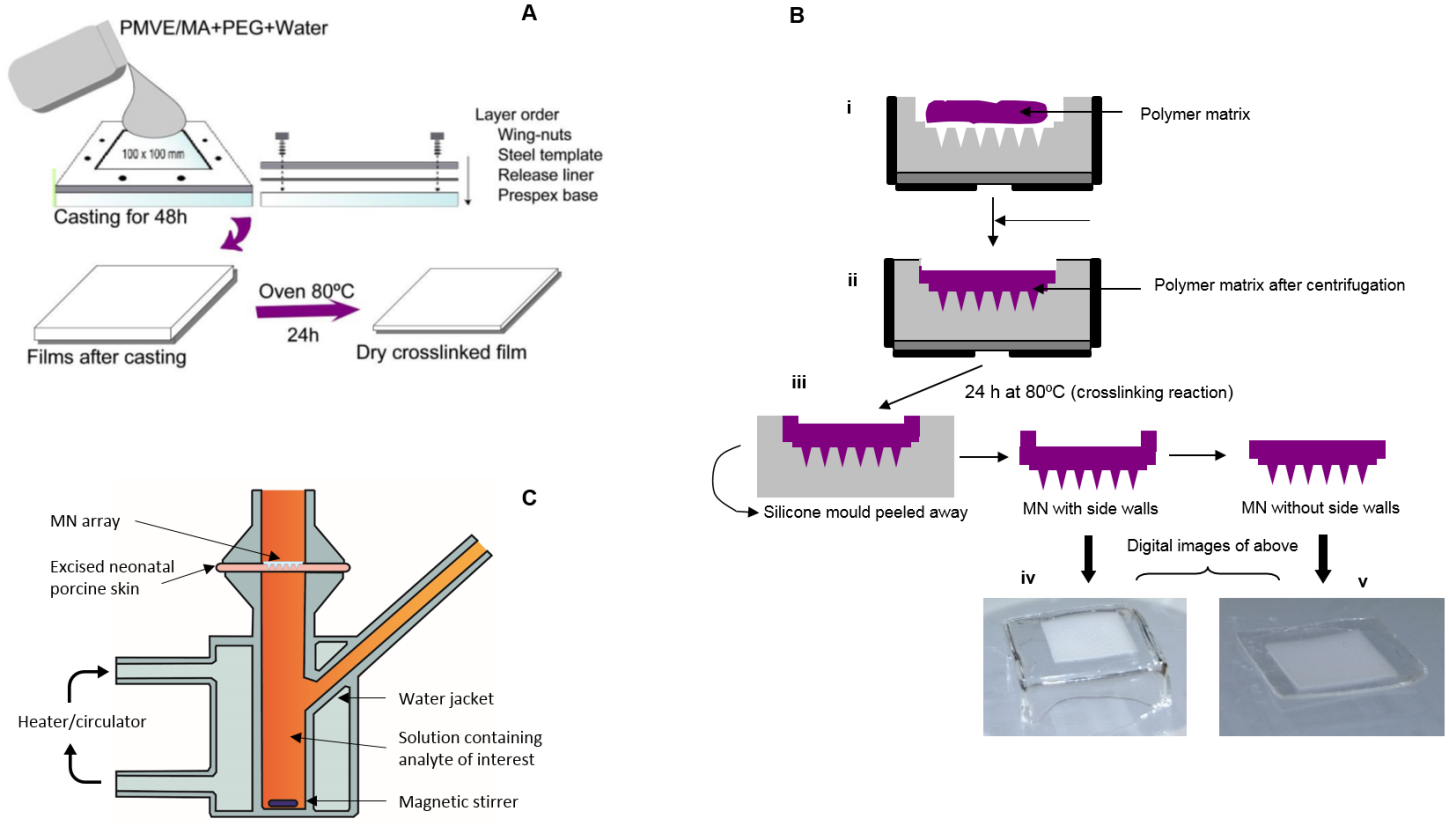
**(A)** Schematic representation of casting and crosslinking of hydrogel film formulations. **(B)** Diagrammatic representation of steps involved in the preparation of polymeric MNs. Polymer matrix was transferred to the silicone mould **(i)**. The mould was centrifuged at 3000 rpm for 15 minutes **(ii)**. Upon drying and heating for 24 h at 80°C to induce ester-based crosslinking, the silicone mould was carefully peeled away from the polymeric MN array and side walls removed using a hot scalpel blade **(iii)**. Digital photograph image of MN with side walls **(iv)**. Digital image of MN after removing the side walls using hot scalpel blade **(v)**. **(C)** Illustration of the modified Franz cell apparatus used to investigate MN uptake of analytes across excised dermatomed neonatal porcine skin *in vitro*.

Aqueous blends containing hydrolysed PMVE/MA (11.1%) and PEG 10,000 (5.6%) were utilized to fabricate MN arrays (19×19 array, 600 μm in height, a base width of 300 μm and interspacing of 50 μm) by using laser-engineered silicone micromould templates [20–22]. MN were crosslinked (esterification reaction) by heating at 80°C for 24 h and sidewalls removed using a heated blade [23–25] (Fig 2). Formulation and mechanical properties of such hydrogel-forming MN have previously been reported [20–22, 26].

### Optical coherence tomography

Optical coherence tomography (OCT) was used to visualise the insertion and swelling of the MN in the skin of healthy human volunteers, as described previously [27].

### *In vitro* extraction and detection using MN arrays

*In vitro* MN-mediated extraction and detection of theophylline, caffeine and glucose was carried out across dermatomed neonatal porcine skin (300–400 μm thickness, shown previously to be a suitable skin model for prediction of *in vivo* performance of MN) [26] by using a modified Franz-cell setup (Fig 2). MN were applied with a force of 11 N per array, as previously reported [22]. *In vitro* fluid uptake by MN arrays was studied using Franz cell receptor chambers thermostated to 37°C and containing varying physiologically relevant concentrations of theophylline, caffeine and glucose in phosphate buffered saline pH 7.4, with MN arrays inserted and removed at defined intervals.

### *In vivo* MN extraction and detection of theophylline

Immediately after application of MN to shaved, hairless skin (Smooth Care^®^, Hair Removal Cream, Boots Company PLC, Nottingham, UK) on the back of each rat, a theophylline solution of 2 mg/mL concentration was administered to the anaesthetised Sprague Dawley^®^ rats *via* oral gavage. Volume of the theophylline solution administered to each rat was based on their individual weight, to achieve a dose of 5 mg/kg or 10 mg/kg. Following application, MN arrays remained *in situ* for 1 h. Blood samples of 200 μl were collected at pre-defined time intervals by lateral tail vein puncture (Microvette^®^ CB 300 LH tubes, Sarstedt AG & Co. Nümbrecht, Germany). All animal experiments throughout this study were conducted according to the policy of the Federation of European Laboratory Animal Science Associations (FELASA) and The European Convention for the protection of vertebrate Animals used for Experimental and Other Scientific Purposes, with implementation of the principle of the 3Rs (replacement, reduction, refinement).

### Human Volunteer Studies

All work undertaken in this study received approval from the School of Pharmacy Research Ethics Committee in Queen’s University Belfast (Study number: 017PMY2012). Fully-informed written consent was obtained from each volunteer prior to their enrolment in the study.

#### Caffeine

Analysis of caffeine uptake by MN was investigated in human volunteers. All subjects abstained from caffeine-containing products for 12 hours prior to undertaking the study, in accordance with study protocol. Each volunteer in the caffeine study received an oral dose of 100 mg (n = 6), in the form of two Proplus^®^ tablets, or 150 mg caffeine (n = 9), in the form of a Starbucks^®^ double-shot espresso coffee [28, 29]. Upon caffeine dosing, 4 MN were applied to each volunteer. Two MN were then removed one hour post-caffeine administration to capture ISF caffeine levels in the 0–1 h window. Following removal of these two initial MN, two further MN were applied to monitor the 1–2 h post-caffeine administration interval. Similarly, upon removal of these MN, a further two MN were applied to monitor the 2–3 h post-administration interval. Three hours post-caffeine dosing those MN which had been in place for the duration of the study (0–3 h) and those which were applied to monitor the 2–3 h interval were removed. For dried blood spot (DBS) analysis of caffeine, 15 μl of blood was spotted, following lancet finger-prick (Unistik^®^ 3 Comfort, Oxfordshire, UK), onto Guthrie^®^ cards, as described previously [30]. All DBS samples were dried for 4 h at room temperature before storage at -80°C prior to analysis. Guthrie cards (Schleicher & Schuell 903^®^) were purchased from Aston Ltd (Oldham, England). The punch used to cut out the DBS was a single 8 mm punch from Darice Inc. (Strongsville, USA).

#### Glucose

Prior to the human volunteer glucose study, each volunteer was asked to fast for a period of at least 2 h. For analysis of glucose uptake by MN, each volunteer received 75 g of glucose powder as an oral dose. MN were applied and removed at identical time points to the caffeine study outline above over a 3 h period. Specifically, MN were applied to monitor ISF glucose levels over 0–1 h, 1–2 h, 2–3 h and 0–3 h intervals post-glucose administration. Furthermore, to assess baseline ISF glucose levels using MN, volunteers had two MN arrays applied 1 h prior to receiving 75 g of glucose powder. Blood glucose levels (BGL) were measured at time of sampling using a glucometer (Accu-Check, Aviva, Roche Ltd., Mannheim, Germany) following lancet finger-prick.

### Extraction of theophylline and caffeine from hydrogel-forming MN arrays

Following *in vitro* and *in vivo* application and removal, all MN were transferred to a sealed, sterile glass vial. Extraction was performed by adding 1 mL HPLC water to each bottle, vortexing for 1 min, then allowing to stand for a further 1 min, at which point all solution in the vial was removed by pipette and transferred to a 1 mL Eppendorf tube. Following centrifugation at 13,000 rpm for 10 min by a SIGMA^®^ 2-16K Centrifuge (SciQuip Ltd. Shropshire, UK) 100 μl of supernatant was removed by pipette and transferred into an auto-sample HPLC vial from which 20 μl was injected into the HPLC column for analysis.

### Preparation and extraction of theophylline from rat blood

For HPLC method validation, blank rat blood samples were obtained and, following centrifuge separation, 20 μl of a theophylline solution of increasing concentration was added to 180 μl of blank plasma. Two consecutive steps were carried out using acetonitrile (ACN) for theophylline extraction. Specifically, following addition of ACN, samples were vortex mixed for 10 min, centrifuged at 13,000 rpm for 10 min at 4°C, and the supernatant collected in disposable glass culture vials. Extract were dried under a stream of nitrogen at 35°C for 50 min using a Zymark TurboVap^®^ LV Evaporator Workstation (Biotage, Uppsala, Sweden) and the residue reconstituted in 200 μl of water, vortex mixed for 3 s and centrifuged at 14,000 rpm at room temperature for 10 min. The supernatant was filtered using a 0.2 μm cellulose acetate filter coupled to a 1 mL syringe and transferred into an auto-sample vial from which 20 μl was injected on to the HPLC column for analysis.

### Preparation and extraction of caffeine from dried blood spots

A stock solution of caffeine was prepared by dissolving 50 mg in 100 mL of HPLC grade water to produce a 500 μg/mL concentration. This was further diluted with water to produce serially diluted working solutions of 160, 120, 80, 60, 40, 20, 10, 5, 2.5 μg/mL, 50 μl of which was added to 950 μl of fresh whole human blood, obtained from volunteers with fully-informed consent. These were then mixed using a Stuart^®^ SB2 fixed speed rotator with Stuart^®^ tube holders (Bibby Scientific, Staffordshire, UK) for 45 min to give final blood concentrations of 8, 6, 4, 3, 2, 1, 0.5, 0.25, 0.125 μg/mL. Drug free blood without caffeine was used as blank. DBS were prepared by pipetting 15 μl of blood onto Guthrie^®^ cards. These were left to dry at room temperature overnight before extraction. Validation was confirmed using spiked concentrations of 7, 2.5, 0.3 μg/mL. For the extraction process, an 8 mm disc was punched out from the centre of DBS samples and transferred to an Eppendorf tube containing 500 μl of a solution of 0.5 μg/mL internal standard (IS) 7-BHT in 100% methanol. The tubes were then vortexed using a Fisons Whirlmixer (Rigal Bennett, Whitley, UK) for 60 min. Following centrifugation at 13,000 rpm for 10 min by a SIGMA^®^ 2-16K centrifuge the supernatant was transferred to a glass vial and evaporated under a stream of nitrogen at 50°C for approximately 60 min using a Zymark TurboVap^®^ LV Evaporator Workstation (Biotage, Uppsala, Sweden). Following evaporation, the residue was reconstituted in 100 μl of HPLC water and dissolved *via* slow vortexing (400 rpm) for 1 min. The resulting solution was then transferred into an auto-sample HPLC vial from which 20 μl was injected onto the HPLC column for analysis.

### Analysis of analyte uptake by MN

Theophylline and caffeine MN samples were quantified using an isocratic HPLC method (Agilent Technologies 1200 Series, Stockport, UK). The separation was performed on an XSelect^®^ HSS C18 (4.6 mm x 100 mm, 3.5 μm) analytical column (Waters Ireland, Dublin) protected with a 20 mm XSelect^®^ guard cartridge of identical chemistry. The column was thermostated at 25°C. The mobile phase consisted of 72.5% 10 mM ammonium acetate buffer and 27.5% methanol at a flow rate of 1.0 mL/min. Sample injection volume was 20 μl. UV detection was performed at 273 nm. The chromatograms obtained were analysed using Agilent ChemStation^®^ Software B.02.01. Least squares linear regression analysis and correlation analysis were performed on the calibration curve produced, enabling determination of the equation of the line, its coefficient of determination and the residual sum of squares (*RSS*). To determine lower limit of detection (*LoD*) and lower limit of quantification (*LoQ*), an approach based on the standard deviation of the response and the slope of the representative calibration curve was employed, as described in the guidelines from ICH [31]. Specifically, the linearity of the method was established by constructing calibration curves for both theophylline and caffeine on three consecutive days. Plots of peak area against analyte concentration were used. The slope, the intercept and the correlation coefficient of each calibration curve were determined. The following equation was then used to estimate the *LoD* for each compound [31]:

$$LoD\;=\;\frac{3.3\cdot \sigma }{S}\;$$

Similarly, the *LoQ* was estimated using the following equation:

$$LoQ\;=\;\frac{10\cdot \sigma }{S}\;$$

where σ = the standard deviation of the response and S = the slope of the calibration curve. The accuracy and precision of the method were determined from analysis of samples at three concentrations representing the low, medium and high portions of the standard curves (LQC, MQC and HQC). The mean accuracy (% RE) and precision (% CV) being within 15% of the actual value, while the *LoQ* which should not deviate by more than 20% [31]. ISF glucose uptake by MN arrays was determined using an Abcam^®^ Assay Kit (Cambridge, UK) and read by a FLUOstar OPTIMA micro-plate reader (Allmendgruenresor, Ortenberg, Germany) at 544 nm and 590 nm excitation and emission wavelengths, respectively.

### Analysis of blood samples

Theophylline and caffeine blood samples were analysed using the same conditions as MN extract analysis, with the exception of variation of the mobile phase to 85% 10 mM ammonium acetate buffer and 15% methanol. The adjustment was made to allow better separation of the chromatographic peaks.

### Statistical analysis

Where appropriate, statistical analyses to compare results were performed using a two-way analysis of variance (ANOVA). In all analyses, *p* < 0.05 denoted statistical significance. Post-hoc statistical comparisons of the means of individual groups were performed using Bonferroni’s post-test. The Statistical Package GraphPad Prism^®^ Version 5.03 (GraphPad Software Inc., CA, USA) was used for all statistical analysis.

## Results and Discussion

MN offer distinct advantages over conventional techniques for TDM. In contrast with conventional blood sampling, MN allow pain-free detection of drug molecules without stimulating the dermal nerves or causing bleeding [5–7]. However, following extraction of biological fluids, future MN technology could exploit internet-based offline analysis, or, potentially incorporate sensors *in situ* to monitor the target drug [32]. Previous studies have proven correlation between the concentrations of drug in ISF with the concentration in blood for a range of drugs [33, 34]. However, to date there has been a paucity of research investigating the potential of MN as a means of drug monitoring [35–37], specifically *via* analysis of compounds in the ISF. As such, this ability of hydrogel-forming MN, as a minimally-invasive mechanism of TDM, is an exciting prospect, as it could negate the use of hypodermic needles, eliminating the pain, bruising and erythema associated with their use [38–41]. Furthermore, risk of infection would also be virtually eliminated by the use of hydrogel-forming MN, since upon intact removal they have insufficient strength in the swollen state to puncture the skin of another patient, thereby negating the risk of needle-stick injuries [39, 40]. In addition, the low cost of polymers and ease of production confers another advantage over traditional hypodermic needles [11]. Hydrogel-forming MN also offer distinct advantages over other MN technologies. Their generic design, self-disabling ability and negligible risk of blockage give them clear benefits over other MN used for monitoring purposes. Importantly, in comparison to dissolving and coated MN which can leave residues behind in patient skin, potentially causing subsequent irritation or sensitisation [42, 43], hydrogel-forming MN leave no detectable polymer residue in patient skin, being removed intact despite softening through uptake of ISF.

### Validation of Analytical Methods

A HPLC method was optimised for the detection of both theophylline and caffeine in both water and plasma samples by simple variation of mobile phase previously used in similar HPLC procedures [44, 45]. The validation of theophylline and caffeine analytical methods was performed in accordance with ICH guidelines [31]. Upon validation, the *LoD* and *LoQ* of theophylline in water was found to be 0.009 and 0.028 μg/mL, respectively, while the *LoD* and *LoQ* values of theophylline in plasma samples was 0.19 μg/mL and 0.57 μg/mL, respectively. As the therapeutic range of theophylline in human plasma is 10–20 μg/mL, the *LoD* and *LoQ* values reported using these methods display a high degree of sensitivity. Furthermore, these values compare favourably to previous HPLC studies analysing theophylline in plasma, in which higher detection and quantification limits around 0.50 μg/mL were reported [46, 47]. Using ICH guidelines, the *LoD* and *LoQ* of caffeine in water was found to be 0.013 μg/mL and 0.039 μg/mL, respectively while, following extraction from DBS, caffeine *LoD* and *LoQ* of 0.24 μg/mL and 0.73 μg/mL, respectively, were obtained. These findings are similar to those previously reported in a study investigating DBS therapeutic caffeine monitoring in neonates, where the *LoQ* was found to be 0.50 μg/mL [48].

For glucose measurements, the fluorescence intensity was measured using the fluorescence plate reader and glucose concentration calculated by comparison with a standard curve for glucose ranging from 0–1600 nmol/mL. The results obtained using this analytical method are in keeping with those reported in similar studies which analysed caffeine [48], theophylline [49] and glucose [50] using HPLC and liquid chromatography-mass spectrometry analysis.

### OCT Analysis

**Fig 3:**
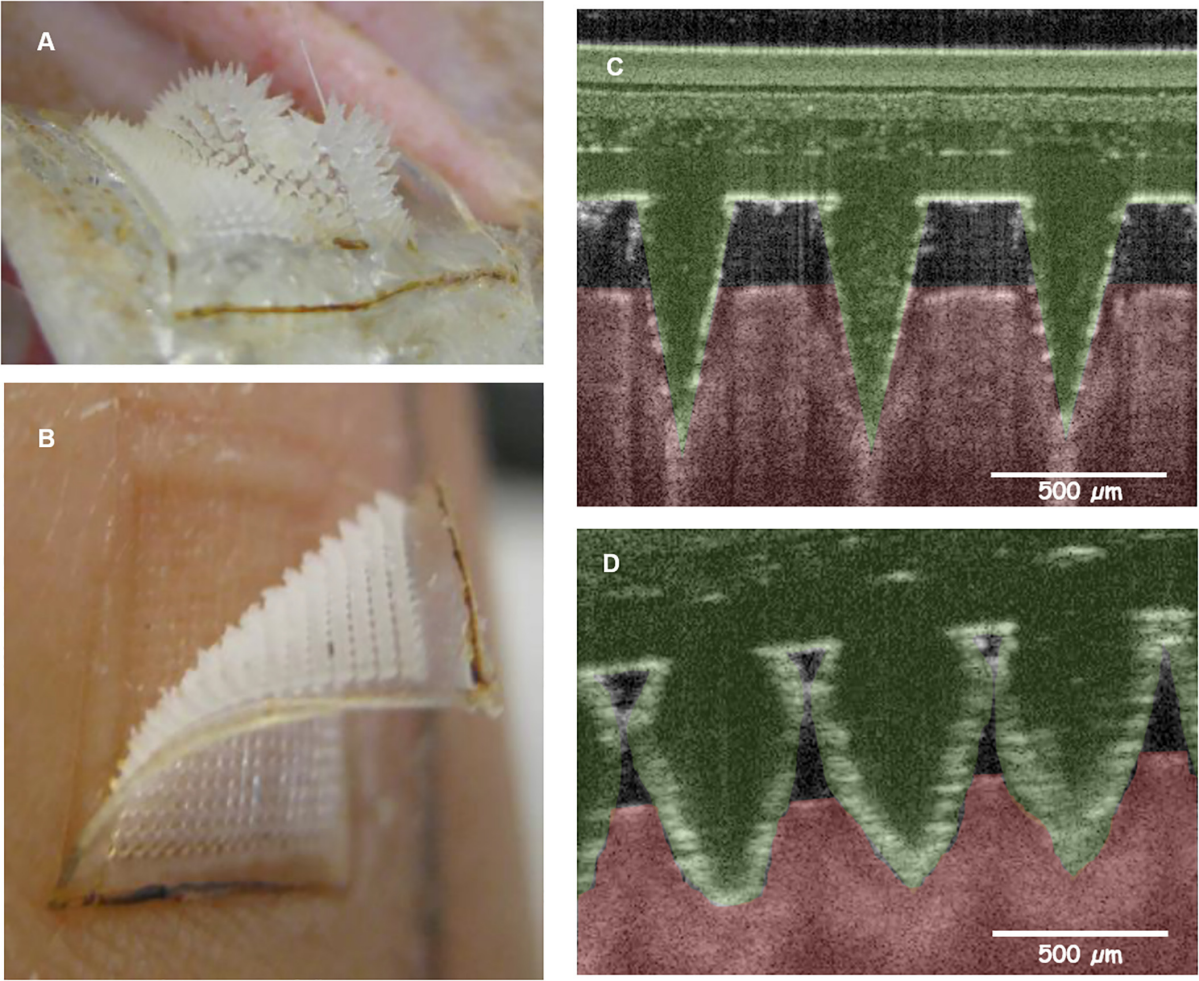
Swollen MN arrays after removal from **(A)** the back of a rat and **(B)** the forearm of a human volunteer, both following 1 h insertion. OCT images showing MN inserted in the forearm of a human volunteer at t = 0 h **(C)** and in the swollen state after 1 h **(D)**.

Previous studies have demonstrated the potential of OCT as a tool to study MN penetration across the *stratum corneum* and to subsequently visualise the dissolving/swelling pattern of MN [22, 51, 52]. The sharpness of our MN allowed them to penetrate the *stratum corneum* of the forearm of human volunteers, as shown in the OCT images at the time of insertion (Fig 3C). However, 1 h later, the MN remained inserted and had swollen with ISF, losing their sharpness (Fig 3D). Despite being soft in the swollen state, MN were always removed intact in all of the *in vivo* studies, demonstrating the inherent safety of our MN technology.

### *In vitro* MN detection of theophylline, caffeine and glucose

**Fig 4:**
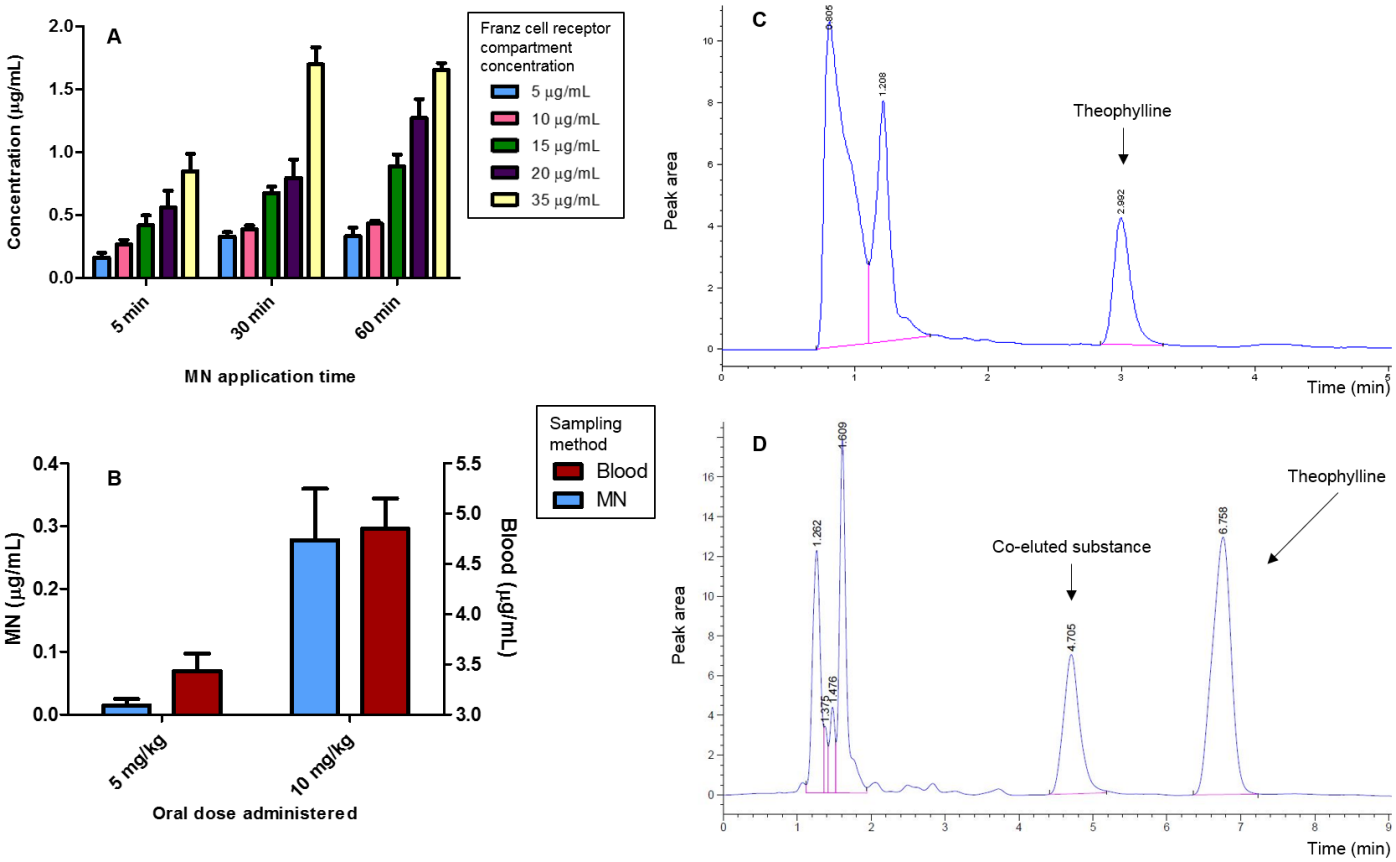
**(A)** Quantities of theophylline (μg) taken up *in vitro* by MN arrays 5, 30 and 60 min after insertion into dermatomed excised neonatal porcine skin mounted on modified Franz cells and bathed on the underside by phosphate buffered saline pH 7.4 thermostated to 37°C and containing defined concentrations of theophylline (Means ± SD, n = 6). **(B)** Quantity of theophylline (μg) taken up *in vivo* by MN arrays inserted into the skin on the back of Sprague Dawley^®^ rats for 1 h after administering theophylline *via* oral gavage at doses of 5 and 10 mg/kg of rat’s body mass (Means ± SD, n = 6). **(C)** Exemplar chromatogram showing HPLC detection of theophylline following extraction from MN after insertion into the skin on a rat’s back for 1 h. Dose administered to rat *via* oral gavage = 10 mg/kg. **(D)** Exemplar chromatogram showing HPLC detection of theophylline from a plasma sample obtained from a rat *via* lateral vein tail puncture 1 h after administration of theophylline (10 mg/kg) *via* oral gavage.

Hydrogel-forming MN are strong enough to puncture the *stratum corneum* when dry and, following skin insertion, they absorb moisture from the skin ISF and swell, allowing substances present in the ISF to permeate into them. Excised neonatal porcine skin has extensively been employed in previous studies as a recognised substitute for human skin [20, 24, 32, 52]. As such, it was selected for *in vitro* use in this study. As shown in Fig 4(A) the amount of theophylline recovered from the MN inserted (5, 30 and 60 min) across neonatal pig skin reflects the increasing concentrations of theophylline (5, 10, 15, 20 and 35 μg/mL) in what is usually the Franz cell receiver compartment. Specifically, after 1 h of MN insertion at the lowest Franz cell concentration (5 μg/mL), a mean recovered concentration of 0.33 μg/mL was reported, whilst at the highest Franz cell concentration (35 μg/mL), a mean theophylline concentration of 1.65 μg/mL was reported. Importantly, these findings also demonstrate that, after only 5 min of insertion, the MN were able to reflect the varying theophylline concentrations in the Franz cell chamber. In particular, concentrations of 0.16 μg/mL ± 0.04 μg/mL and 0.85 μg/mL ± 0.14 μg/mL were detected for the lowest and highest concentrations of theophylline, respectively, after 5 min. These results are in line with work by Kendall *et al*., where MN coated with capture molecules were able to detect the presence of biomarkers within 10 min of insertion [53]. In the present study, the noted variability observed in the release measurements for MN inserted into neonatal porcine skin for 5 min could potentially reflect slight differences in the rates of initial diffusion of fluid from the Franz cell into the hydrogel matrix [54, 55], since swelling is necessary for substance capture.

Statistical analysis of values shown in Fig 4(A) confirm a highly significant difference (*p* < 0.01) in theophylline levels detected after 5, 30 and 60 min at all concentrations, with the exception of the 15 μg/mL against 20 μg/mL Franz cell receptor compartment. Furthermore, there was a very highly significant difference (*p* < 0.001) at the 5 min time point when theophylline levels detected from the 5 μg/mL and 10 μg/mL Franz cell receptor compartment were compared with levels from the 35 μg/mL compartment. Similarly, there was a significant difference (*p* < 0.05) in theophylline levels detected at the 5 min time point when the 15 μg/mL and 35 μg/mL concentrations were compared. These findings suggest that, for theophylline TDM, shorter periods of MN insertion may actually be preferable, since any difference in concentration can be detected at a relatively early stage. This, in turn, could facilitate patient compliance and, importantly, provide clinically-relevant drug or biomarker data to the clinician in a timely fashion.

**Fig 5:**
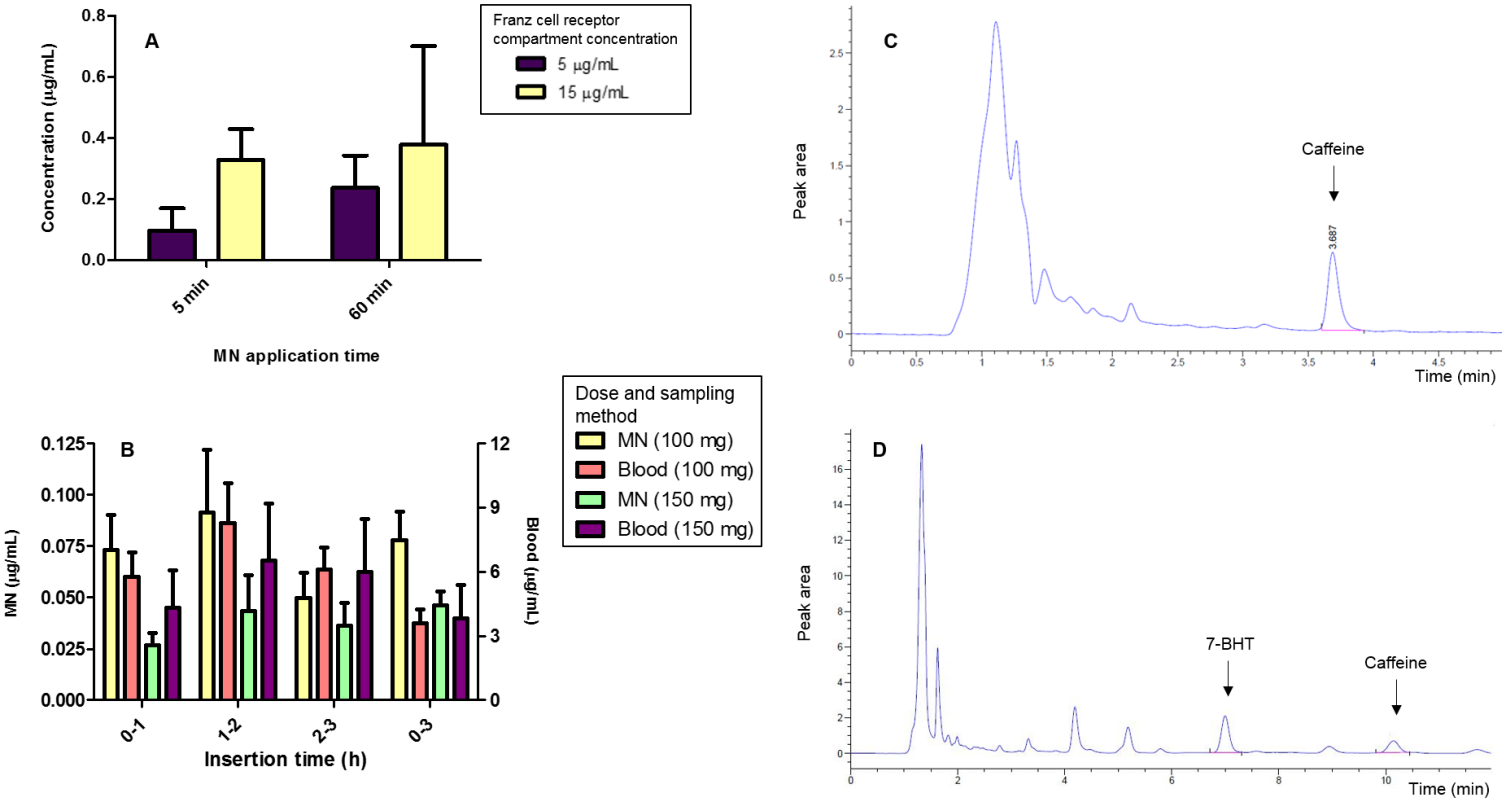
**(A)** Quantity of caffeine (μg) taken up *in vitro* by MN arrays 5 and 60 min after insertion into dermatomed excised neonatal porcine skin mounted on modified Franz cells and bathed on the underside by phosphate buffered saline pH 7.4 thermostated to 37°C and containing defined concentrations of caffeine (Means ± SD, n = 6). **(B)** Comparison of caffeine (μg) detected in plasma versus levels detected from MN arrays inserted in the forearm of human volunteers at defined time points (0–1, 1–2, 2–3 and 0–3 h) following administration of defined caffeine doses (Mean ± SD, n = 9). **(C)** Exemplar chromatogram showing HPLC detection of caffeine following extraction from MN after insertion in the forearm of a human volunteer for 1 h. Dose administered = 100 mg oral caffeine in the form of two Proplus^®^ tablets. **(D)** Exemplar chromatogram showing HPLC detection of caffeine from a plasma sample obtained from a human volunteer 1 h after the administration of 100 mg of caffeine. 7-BHT was used as an internal standard.

The potential of MN as a means of minimally-invasive means of TDM and diagnosis has recently become the focus of considerable interest [32, 56, 57]. Although previous studies have detected certain compounds using MN in combination with other techniques [57], no study has demonstrated the ability of MN alone to both detect and quantify analytes of clinical interest, thereby facilitating both diagnosis and TDM. In addition to theophylline, this study investigated the ability of hydrogel-forming MN to detect caffeine *in vitro*, using the previously-described Franz cell setup. Fig 5(A) shows the extracted caffeine levels from MN inserted into neonatal porcine skin at defined time points and Franz-cell concentrations. As expected, caffeine levels detected from 5 and 60 min MN insertion times appropriately reflect the different concentrations present in the respective Franz cell compartments, with mean caffeine levels detected from the 15 μg/mL Franz cells being higher than mean caffeine levels from the 5 μg/mL Franz-cells at both time points. As Fig 5(A) clearly shows, we have demonstrated that our hydrogel-forming MN possess the ability to uptake sufficient ISF after only 5 min insertion time to allow quantification of caffeine. Despite no statistically significant difference (*p* > 0.05) identified between *in vitro* caffeine levels detected at 5 and 60 min time points in the 5 μg/mL and 15 μg/mL Franz-cell receptor compartments, the levels detected at both time points had increased. Specifically, the mean concentration of 0.10 μg/mL obtained from the 5 μg/mL Franz-cell after 5 min had increased to 0.23 μg/mL after 60 min, while the mean concentration obtained from the 15 μg/mL Franz cell increased from 0.33 μg/mL to 0.38 μg/mL over the same application time. With respect to MN-mediated TDM, specifically relating to drugs with a narrow therapeutic index, these findings further suggest that hydrogel-forming MN may have the potential to rapidly differentiate between high and low drug concentrations. Crucially, this ability to distinguish between a therapeutic drug concentration and a sub-therapeutic or toxic concentration could allow hydrogel-forming MN to provide invaluable guidance on dosing of drugs with a narrow therapeutic range in which rapid measurement is recognised as a major factor in optimising clinical outcome [58].

**Fig 6:**
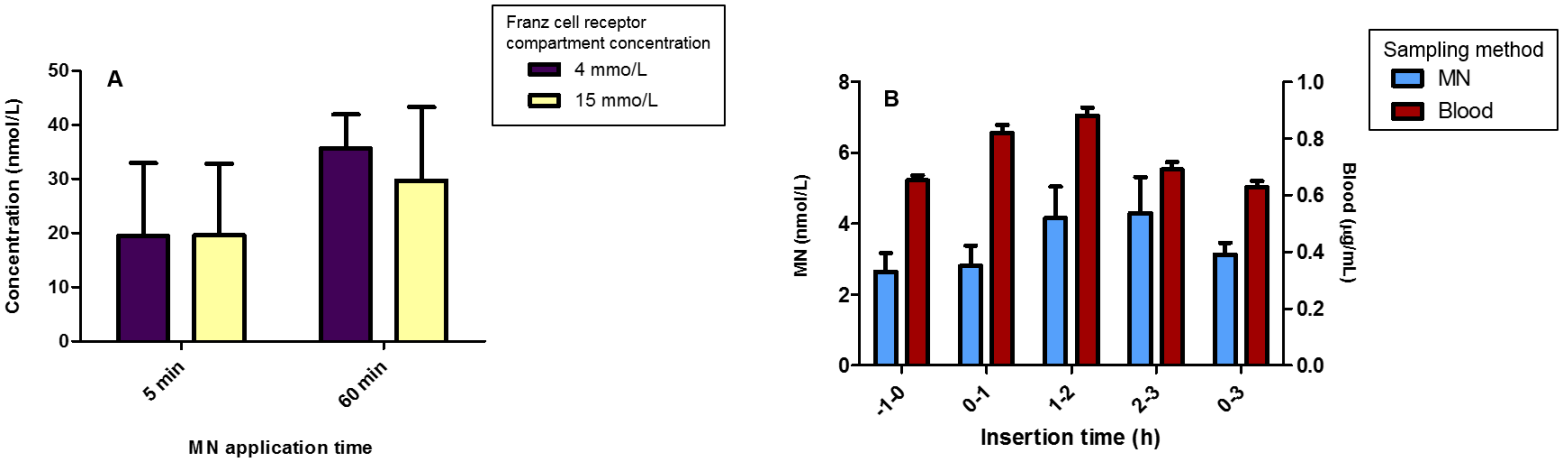
**(A)** Quantity of glucose (μg) taken up *in vitro* by MN arrays 5 and 60 min after insertion into dermatomed excised neonatal porcine skin mounted on modified Franz cells and bathed on the underside by phosphate buffered saline pH 7.4 thermostated to 37°C and containing defined concentrations of of glucose (Means ± SD, n = 6). **(B)** Comparison of glucose (μg) detected in plasma versus levels detected from MN arrays inserted in the forearm of human volunteers at defined time points preceding (-1-0 h) and following (0–1, 1–2, 2–3 and 0–3 h) oral administration of 75 g of glucose (Means ± SD, n = 9).

Similar to the above studies, *in vitro* analysis was performed to investigate the ability of the hydrogel-forming MN to extract glucose through neonatal porcine skin using a modified Franz-cell setup, as described previously. As shown in Fig 6(A), the mean detected glucose concentration after 5 min from the Franz cell containing 4 mmol/L was 19.46 nmol/L. Although not statistically significant (*p* > 0.05), the mean detected glucose concentration after 60 minutes application had approximately doubled to 35.67 nmol/L. These results successfully demonstrate the *in vitro* ability of hydrogel-forming MN to detect glucose in neonatal porcine skin. The promising nature of the obtained results, which quantify glucose after only 5 minutes *in vitro*, led us to proceed to human volunteer studies.

This study demonstrates the ability of hydrogel-forming MN to detect and quantify theophylline, caffeine and glucose following only 5 min insertion in neonatal porcine skin. In addition, it demonstrates the ability of hydrogel-forming MN arrays to rapidly detect various drugs with only 5 min exposure in a fresh water solution as opposed to the overnight exposure required in previous studies to detect glucose and sodium ions from poly(vinyl alcohol) hydrogels [59]. Notably, this previous study involved a time-consuming two-step approach, in which volunteer forearms were stamped with a plastic MN. This was then followed by application of a hydrogel patch placed on the stamped area, with the aim of accumulating ISF glucose [59]. Significantly, in this present study, hydrogel-forming MN were able to differentiate between *in vitro* theophylline concentrations of 5 μg/mL and 15 μg/mL after 60 minutes MN application (*p* < 0.001) and 5 μg/mL and 35 μg/mL after only 5 minutes application time (*p* < 0.001) but without the assorted problems related to needle insertion and associated processing [38–41].

### *In vivo* MN detection of theophylline

Theophylline is a xanthine bronchodilator typically used in the treatment of asthma and chronic obstructive pulmonary disease [60]. It possesses a narrow therapeutic range, with a plasma concentration of 10–20 mg/L usually providing a satisfactory therapeutic response. Importantly, the desired therapeutic range in neonates is typically lower at 5–10 mg/L [61, 62]. As the metabolism of theophylline is subject to several variable factors, plasma concentration often requires monitoring in individual patients to ensure both optimal clinical response and avoidance of undesirable toxic effects [63, 64]. Typical effects of toxic levels of theophylline (> 25 mg/L) in both adults and neonates include tachycardia and central nervous system excitation, whilst in severe cases, potentially fatal theophylline-associated seizures can occur [65]. Previous studies in rats have reported a linear pharmacokinetic profile of theophylline for doses not exceeding 10 mg/kg of rat body mass [66]. Therefore, this was chosen as the higher dose administered to the rats in this study. The average concentration of theophylline extracted from MN inserted for 1 h in the rats’ back, and the average theophylline concentration extracted from blood between 0 h (pre-theophylline administration) and 1 h are shown in Fig 4(B). This allowed the comparison of mean blood analyte values over a 1 h time period and total analyte extracted using hydrogel-forming MN in the 1 h time period. The insertion period employed was chosen conservatively in order to ensure sufficient uptake of fluid for accurate theophylline quantification, thus minimising the number of rats used, bearing in mind the requirements of the 3 Rs.

Theophylline was successfully extracted by MN arrays and subsequently detected from the ISF, as depicted in the chromatogram obtained from the extraction of theophylline from an individual MN array (Fig 4C). The highest theophylline concentration detected from a hydrogel-forming MN from a rat dosed with 10 mg/kg was of 0.363 μg/mL, whilst a maximum of 0.063 μg/mL was detected from a rat dosed with 5 mg/kg theophylline. Statistical analysis of the values shown in Fig 4(B) demonstrated that, following oral theophylline doses of 5mg/kg and 10mg/kg, a very highly significant difference (*p* < 0.001) in theophylline levels between both concentrations administered is detectable *via* both blood and MN. These findings demonstrate that hydrogel-forming MN have the *in vivo* ability to differentiate between significantly different (*p* < 0.001) ISF concentrations of a drug with a narrow therapeutic range, thus raising the very real possibility of hydrogel-forming MN as a means to determine whether a drug with a narrow therapeutic range is within its therapeutic window.

Notably, no theophylline was detected from MN-free baseplates (prepared using identical formulae to the MN, as described in Fig 2A) applied to the rats’ backs. This suggests that the swelling of MN was due to uptake of ISF containing theophylline rather than any theophylline potentially present in rat perspiration. In addition, our polymeric MN systems were removed macroscopically intact after skin insertion, leaving no discernible polymer behind.

### Human volunteer studies for caffeine

With the aim of investigating the ability of hydrogel-forming MN to detect caffeine in ISF, we applied MN to the ventral forearm of healthy male and female human volunteers with no existing skin conditions. Fig 3(B) shows MN swelling after 1 h insertion in a human volunteer, demonstrating that hydrogel-forming MN are able to imbibe ISF and swell over a relatively short period of time *in vivo*. This was in line with what was seen in the rat studies. The levels of caffeine detected by both MN and DBS are shown in Fig 5(B). As with the *in vivo* theophylline study, this allowed the comparison of mean blood analyte values and total analyte extracted using hydrogel-forming MN over the same time period. As demonstrated, our novel hydrogel-forming MN were able to quantify caffeine taken up by MN arrays at each time point. Fig 5(C) shows a chromatogram demonstrating caffeine detection *via* hydrogel-forming MN using the above method, and as outlined in Fig 1. Significantly, our MN readily detected caffeine after only 1 h post-consumption. The mean detected level of 73.05 μg/mL demonstrates that TDM using hydrogel-forming MN is a feasible concept in human subjects and can provide rapid analyte quantification soon after administration. These findings further emphasise the advantages of our hydrogel-forming MN technology, which include ease of sampling and storage, reduced problems associated with time-consuming analysis of blood-based matrices and the avoidance of hypodermic-needle use, when compared with traditional blood sampling. The highest mean concentration of caffeine detected using our MN was 91.31 μg/mL over the period from 1 to 2 h post-consumption of 100 mg Proplus^®^. This result is similar to previous findings which reported caffeine absorption from the small intestines within 45 minutes of consumption and peak blood concentration occurring after 1–2 h[67]. Furthermore, in keeping with the pharmacokinetics of caffeine, MN arrays applied during the 2–3 h interval post-caffeine consumption demonstrated the lowest mean caffeine level of 49.56 μg/mL, suggesting that, by this time, the absorbed caffeine is likely to be undergoing hepatic metabolism to its three primary metabolites; paraxanthine, theobromine and theophylline. Of further interest is the level of caffeine detected in the MN arrays applied continuously for 3 h. As shown in Fig 5(B), these arrays detected a much lower level of caffeine than the MN applied in the 1–2 h post-caffeine interval and a similar level to that detected in the 0–1 h interval. A possible explanation for this is that the detected level in the MN reflects the established pharmacokinetics of caffeine. Specifically, after 3 h, significant hepatic caffeine metabolism has already occurred and, as a result of their continuous application, these MN arrays may have reached an equilibrium with the ISF, resulting in a lower detected level of caffeine than that reported in the peak interval of 1–2 h. Given these findings it would therefore, be appropriate, as well as advantageous, to apply the MN arrays for a maximum period of 1 h.

In addition to 100 mg caffeine as Proplus^®^, Fig 5(B) also shows caffeine levels detected from both MN and DBS following volunteer consumption of a Starbucks^®^ double-shot espresso coffee. Interestingly, despite a purported caffeine content of 150 mg [28, 29], both MN and DBS sampling following coffee consumption demonstrated caffeine levels lower than those obtained following administration of 100 mg caffeine (as Proplus^®^) at identical time points. Despite reference sources indicating a caffeine content of 150 mg present in the coffee, the results would indicate this not to be the case. It is possible that each coffee had a different caffeine content, due to the variability associated with human preparation. Alternatively, the formulation of the coffee itself could have impaired absorption. However, the latter would appear unlikely, as coffee, being in liquid form would, in all likelihood, actually improve absorption.

As shown in Fig 5(D), caffeine was also readily detected after 1 h in DBS sampled concurrently with MN. Caffeine detected *via* DBS is reported at higher concentrations than MN concentrations. However, it is notable that, whilst the values detected are statistically different due to the higher detected DBS concentrations (*p* > 0.05), the pharmacokinetic profile of MN and DBS sampling displays general agreement over the 0–1 h and 1–2 h time intervals. As shown in Fig 5(B), both MN and DBS sampling show peak caffeine levels in the 1–2 h interval post-caffeine consumption. However, as above, due to the increased concentration of caffeine detected using the DBS method, the values reported are statistically different (*p* > 0.05). Similarly, MN and DBS sampling following the purported 150 mg dose of caffeine both indicate a lower detected caffeine level than that following 100 mg caffeine (as Proplus^®^). Despite the differences in concentrations reported between DBS and MN sampling, the similar concentration profile demonstrated for DBS and MN displayed in Fig 5(B) suggests that, with a validated mathematical method, it may well be possible to correlate the detected MN concentration to that detected from DBS sampling. Such a conversion could, therefore, make hydrogel-forming MN a realistic means of TDM.

Although previous studies have studied the collection of ISF using a variety of MN designs [68–70], to the best of our knowledge, this is the first MN study to demonstrate detection and quantification of a model drug substance (caffeine) in human subjects following oral administration. The simplicity of the methodology employed also overcomes problems associated with other methods employed for ISF-mediated TDM, such as RI and CM, while Fig 3(B) clearly demonstrates the minimally-invasive nature of the technology. These findings unequivocally demonstrate the potential of our hydrogel-forming MN arrays as a means of simple and rapid TDM, whilst avoiding the aforementioned problems associated with conventional blood sampling [32]. In addition, the analytical process illustrates how TDM using hydrogel-forming MN could be incorporated into a conventional clinical laboratory setting, thus further emphasising the potential of our novel MN technology as a means monitoring clinically-important drugs and biomarkers.

### Human Volunteer Studies for Glucose

Despite numerous proposed methods for ISF extraction from the skin, including RI, sonophoresis and laser ablation for creation of micropores, Glucowatch^®^, (Cygnus Redwood City, CA) remains the only method endorsed by the US FDA. However, the Glucowatch^®^ system uses an electric current for ISF extraction, which can cause pain, irritation and damage to the treated skin [71]. Therefore, the development of a minimally-invasive or non-invasive, and pain free method remains the main focus in production of new systems for glucose monitoring.

Over recent years, MN have become one of the most promising minimally-invasive and pain-free approaches for ISF glucose extraction [71]. However, lack of sufficient data regarding their practical application has led to a need to establish if ISF glucose can be extracted using MN and, if so, can the extracted glucose concentration be quantified and/or correlated to that found in blood [71]. To date, previous studies have investigated both dissolving and hollow MN as means of minimally-invasive glucose monitoring [57, 71]. However, unlike these previous studies, which based their conclusions on a two-step process in a rat model and uptake of simulated ISF by hollow MN in a petri dish respectively [57,71], our study focussed solely on *in vivo* glucose ISF monitoring in human volunteers. Specifically, we evaluated the potential of MN-mediated transdermal glucose monitoring in healthy volunteers and compared the obtained results with those from finger-prick samples using the Accu-Check^®^ Aviva glucometer. Glucose concentrations at the sampled time points are shown in Fig 6(B). The highest mean BGL was 7.89 nmol/L nmol/L detected 1 h following ingestion of 75 g of glucose, while the highest mean glucose concentration extracted from MN was 4.29 nmol/L detected after 3 hours. This 2 hour difference between MN levels and BGL may be explained by a lag-time phenomenon, which has been previously described in several studies [72] and would explain the statistical difference between MN and finger-prick BGL at identical time points (*p* > 0.05). Briefly, the lag-time is due to the delay in transport of glucose from plasma, *via* the vasculature, to the interstitial space. It is also likely that there is a high degree of variability between individuals, due to individual metabolism. Previous studies in both diabetic and non-diabetic individuals have reported this effect may take between 4 to 50 minutes to occur [73]. The appearance of the peak BGL after 2 hours is in keeping with other studies that investigated the glucose profile of healthy volunteers following an oral glucose tolerance test (OGTT) [74]. Despite the longer application time, the mean detected glucose from the MN applied for a full 3 hours was 3.13 nmol/L, lower than the concentration detected after 2 hours. This may be explained by the equilibrium effect between ISF and MN, as outlined above for caffeine.

These findings demonstrate that ISF glucose measurements using hydrogel-forming MN tracked the fluctuation in BGL, with an approximate 1 hour lag time. As discussed previously, this may allow development of mathematical correlation or algorithm to compensate for the time difference between BGL and ISF glucose concentrations at a given time point [75], which in turn could facilitate rapid insulin delivery in conjunction with existing technologies [76]. In addition, several other factors may influence differences between ISF glucose and BGL. Firstly, the altered pharmacokinetic and pharmacodynamic parameters of glucose resulting from its movement in and out of the ISF during the period of MN application [77]. Secondly, the small ISF volume contained within the epidermis may delay migration of ISF glucose in a sufficient quantity to imbibe the hydrogel-forming MN [72]. Despite these challenges, this study demonstrates the exciting potential of hydrogel-forming MN to extract glucose from ISF in human subjects *in vivo*. Therefore, future work will build upon the findings in this study prior to clinical use. In addition, studies will be conducted in a larger population to facilitate statistical correlation of ISF glucose values to those which are present in volunteer blood samples.

## Conclusion

This study has shown, for the first time, the potential of hydrogel-forming MN as a tool for diagnosis, *via* biomarker detection, and TDM. We have demonstrated the ability of hydrogel-forming MN to detect and quantify three different analytes from ISF *in vivo*. Hydrogel-forming MN investigated in this study offer numerous advantages over conventional techniques used for TDM. Firstly, in contrast with hypodermic needles, MN allow sampling and detection of analytes in a minimally-invasive manner, without causing any bleeding or pain. Secondly, hydrogel-forming MN eliminate the risk of needle-stick injury, leading to infection, as previously discussed. In addition, in the Developing World, MN could dramatically reduce the need for medically-trained personnel, and their associated cost, by minimising the need for hypodermic needle use. Furthermore, premature neonates in all settings could also benefit from this minimally-invasive technology, as they are routinely treated with multiple drugs, many of which require frequent TDM. Given their reduced blood volume, and the accompanying risk of iatrogenic anaemia with conventional blood-based monitoring of drugs and biomarkers, hydrogel-forming MN could provide an ideal alternative.

Hydrogel-forming MN offer numerous advantages over other types of MN. Despite their softness in the swollen state, hydrogel-forming MN were removed intact in all *in vivo* studies, without leaving detectable polymeric residue in the skin. This minimises the risk of skin irritation associated with solid and hollow MN, given that no signs of irritation were observed in the rats’ backs or in the forearms of human volunteers as a consequence of the application of MN during the various studies. Furthermore, the risk of blockage of the central conduit of a hollow MN would be obviated with the use of hydrogel-forming MN, which create an unblockable conduit for ISF uptake from the viable skin. In addition, the use of coated MN, despite their high specificity for a targeted molecule, could possibly be associated with some disadvantages. In particular, some of the coating of the MN could accidentally become detached, delivering an antibody or exogenous compound which was not expected to be delivered and could possibly cause an undesired immune or inflammatory response.

The primary objective of this study was to determine if analyte detection *via* ISF was possible using hydrogel-forming MN and, in addition, to discover whether hydrogel-forming MN could differentiate between high and low concentrations of analyte in ISF. The results of this study clearly demonstrate that hydrogel-forming MN could be used as a minimally-invasive means of TDM and biomarker detection. The next step in development of this technology will seek to demonstrate the ability to differentiate between high and low concentrations of a wide range of therapeutic agents, with a particular focus on compounds with a narrow therapeutic window. Equally, biomarker detection offers a substantial target for our future clinical research. The success of hydrogel-forming MN as a means of minimally-invasive monitoring, and detection of compounds of clinical interest, will open up a wide field of therapeutic opportunity. Toxic or sub-therapeutic concentrations of therapeutic compounds could be rapidly identified, without the need for hypodermic needles or heel-prick sampling, and subsequent dosing adjusted accordingly. Detection of widely tested biomarkers, such as C-reactive protein or brain natriuretic peptide, could occur at home without the need for *in situ* medical personnel and such MN could incorporate ‘lab-on-a-chip’ technology, allowing remote access to the information by an attending clinician prior to the patient arriving at hospital. This could also avoid the need for unnecessary hospital attendance. Similarly, in the developing world, biomarker detection by hydrogel-forming MN would allow for diagnosis of a wide range of infectious diseases, most of which have specifically-associated biomarkers, again negating the need for application by expensively trained medical personnel, whilst in western medicine, hydrogel-forming MN technology would allow detection of drugs of abuse without the need for problematic blood sampling. However, prior to the widespread use of hydrogel-forming MN as a means of minimally-invasive monitoring, further evidence of efficacy in clinical trials is required, as are investigations to determine whether they possess the ability to detect a suitable range of drug compounds and biomarkers. Accordingly, our present focus is twofold. Firstly, as outlined above, we aim to investigate a range of compounds suitable for rapid detection using hydrogel-forming MN. In addition, we are also aiming to keep MN application time to a minimum. As we have demonstrated here, within 5 minutes, our novel MN arrays can uptake sufficient ISF in to allow analyte detection *in vitro*. If this application time can be replicated in human volunteers, for a wide range of clinically-relevant compounds, then our hydrogel-forming MN will provide a TDM snapshot currently only obtained *via* invasive blood sampling methods.

## References

[1] Schumacher GE. Introduction to therapeutic drug monitoring In: Schumacher GE, editor. Therapeutic drug monitoring. Norwalk (CT): Appleton & Lange, 1995: 1–17

[2] Aronson JK, Hardman M. ABC of monitoring drug therapy: measuring plasma drug concentrations. BMJ 1992; 305:1078–1080. 1467691 10.1136/bmj.305.6861.1078 PMC1883634

[3] Leboulanger B, Guy RH, Delgado-Charro MB. Reverse iontophoresis for non-invasive transdermal monitoring. Physiol Meas. 2004; 25:R35–50. 15253111 10.1088/0967-3334/25/3/r01

[4] Brunner M, Derendorf H. Clinical microdialysis: Current applications and potential use in drug development. Trends in Analytical Chemistry 2006; 25:674–680.

[5] Gill H, Denson D, Burris B, Prausnitz M. Effect of microneedle design on pain in human volunteers. Clin J Pain. 2008; 24:585–94. 10.1097/AJP.0b013e31816778f9 18716497 PMC2917250

[6] Haq M, Smith E, John D, Kalavala M, Edwards C, Anstey A. Clinical administration of microneedles: skin puncture, pain and sensation. Biomed. Microdevices. 2009; 11:35–47. 10.1007/s10544-008-9208-1 18663579

[7] Kaushik S, Hord A, Denson D, McAllister D, Smitra S, Allen M. Lack of pain associated with microfabricated microneedles. Anesth. Analg. 2001; 92:502–504. 11159258 10.1097/00000539-200102000-00041

[8] Trzebinski J, Sharma S, Moniz A, Michelakis K, Zhang Y. Microfluidic device to investigate factors affecting performance in biosensors designed for transdermal applications. Lab. Chip. 2012; 12:348–352. 10.1039/c1lc20885c 22130554

[9] Windmiller J, Valdes Ramirez G, Zhou N, Zhou M, Miller P. Bicomponent Microneedle array biosensor for minimally-invasive glutamate monitoring. Electroanalysis. 2011; 23:2302–2309.

[10] Windmiller J, Zhou N, Chuang M, Valdes-Ramirez G, Santhosh P, Miller P. Microneedle array-based carbon paste amperometric sensors and biosensors. Analyst. 2011; 136:1846–1851. 10.1039/c1an00012h 21412519

[11] Corrie S, Fernando G, Crichton M, Brunck ME, Anderson CD, Kendall MA. Surface-modified microprojection arrays for intradermal biomarker capture, with low non-specific protein binding. Lap Chip. 2010; 10:2655–2658. 10.1039/c0lc00068j 20820632

[12] Jenkins D, Corrie S, Flaim C, Kendall M. High density and high aspect ratio solid micro-nanoprojection arrays for targeted skin vaccine delivery and specific antibody extraction. RSC Adv. 2012; 2:3490–3495.

[13] Muller DA, Corrie SR, Coffey J, Young PR, Kendall MA. Surface modified microprojection arrays for the selective extraction of the dengue virus NS1 protein as a marker for disease. Anal Chem. 2012; 84:3262–3268. 10.1021/ac2034387 22424552

[14] Bhargav A, Muller DA, Kendall MA, Corrie SR. Surface modifications of microprojection arrays for improved biomarker capture in the skin of live mice. ACS Appl Mater Interfaces. 2012; 4:2483–2489. 10.1021/am3001727 22404111

[15] Donnelly RF, Thakur RRS, Garland MJ, Migalska K, Majithiya R, McCrudden CM et al. Hydrogel-forming Microneedle arrays for enhanced transdermal drug delivery. Adv. Funct. Mater. 2012a; 22:4879–4890. 23606824 10.1002/adfm.201200864 PMC3627464

[16] Donnelly RF, Woolfson AD, McCarron PA, Morrow DIJ, Morrissey A (2007). Microneedles/Delivery Device and Method. British Patent Application No 0718996.2. Filed September 28th 2007. International publication No WO2009040548.

[17] Donnelly RF, Bell SEJ, Jones DS, McCoy CP (2010). Microneedle-mediated enhanced Raman therapeutic drug monitoring (EP/H021647/1). Engineering and Physical Sciences Research Council (EPSRC) £327,441. http://gow.epsrc.ac.uk/NGBOViewGrant.aspx?GrantRef=EP/H021647/1.

[18] Donnelly RF, McCoy CP, McCarthy HO, McElnay JC (2012). Microneedle-enhanced neonatal therapeutic drug monitoring (GN2024). Action Medical Research (AMRC) £94,154. http://www.action.org.uk/our-research/neonatal-care-new-way-monitor-drug-levels-could-spare-babies-painful-blood-tests.

[19] Romanyuk AV, Zvezdin VN, Samant P, Grenader MI, Zemlyanova M, Prausnitz MR (2014). Collection of analytes from microneedle patches. Analytical Chemistry, 86, 10520–10523. 10.1021/ac503823p 25367229 PMC4222632

[20] Migalska K, Morrow DI, Garland MJ, Thakur R, Woolfson AD, Donnelly RF. Laser-engineered dissolving microneedle arrays for transdermal macromolecular drug delivery. Pharm Res. 2011; 28:1919–30. 10.1007/s11095-011-0419-4 21437789

[21] Donnelly RF, Majithiya R, Thakur RRS, Desmond DIJ, Garland MJ, Demir YK et al. Design, optimization and characterisation of polymeric microneedle arrays prepared by a novel laser-based micromoulding technique. Pharm. Res. 2011; 28:41–57. 10.1007/s11095-010-0169-8 20490627 PMC3016610

[22] Donnelly RF, Garland MJ, Morrow DI, Migalska K, Singh TR, Majithiya R et al. Optical coherence tomography is a valuable tool in the study of the effects of microneedle geometry on skin penetration characteristics and in-skin dissolution. J. Control. Release. 2010; 147:333–341. 10.1016/j.jconrel.2010.08.008 20727929

[23] Thakur RRS, McCarron PA, Woolfson AD, Donnelly RF. Investigation of swelling and network parameters of poly (ethylene glycol)-crosslinked poly (methyl vinyl ether-co-maleic acid) hydrogels. Eur Polym J. 2009; 45:1239–1249.

[24] Thakur RRS, Woolfson AD, Donnelly RF. Investigation of solute permeation across poly (ethylene glycol)-crosslinked poly (methyl vinyl ether-co-maleic acid) hydrogels. J Pharm Pharmacol. 2010; 62:829–837. 10.1211/jpp.62.06.0003 20636870

[25] Garland MJ, Thakur RRS, Woolfson AD, Donnelly RF. Electrically-enhanced solute permeation across poly (ethylene glycol)—crosslinked poly (methyl vinyl ether—co- maleic acid) hydrogels: Effect of hydrogel crosslink density and ionic conductivity. Int J Pharm. 2008; 406:91–98. 10.1016/j.ijpharm.2011.01.002 21236323

[26] Donnelly RF, Morrow DI, Singh TR, Migalska K, McCarron PA, O'Mahony C et al. Processing difficulties and instability of carbohydrate microneedle arrays. Drug Dev Ind Pharm. 2009; 35:1242–54. 10.1080/03639040902882280 19555249 PMC2900182

[27] Donnelly RF, Mooney K, McCrudden MT, Vicente-Pérez EM, Belaid L, González-Vázquez P et al. Hydrogel-forming microneedles increase in volume during swelling in skin, but skin barrier function recovery is unaffected. J Pharm Sci. 2014; 103:1478–86. 10.1002/jps.23921 24633895 PMC4119956

[28] Starbucks Coffee Company. Available: http://www.starbucks.com/menu/catalog/product?drink=espresso#view_control Accessed 2015 Mar 24.

[29] Caffeine Informer. Available at: http://www.caffeineinformer.com/the-complete-guide-to-starbucks-caffeine Accessed 2015 Mar 24.

[30] Hawwa AF, Albawab A, Rooney M, Wedderburn LR, Beresford MW, McElnay JC. A novel dried blood spot-LCMS method for the quantification of methotrexate polyglutamates as a potential marker for methotrexate use in children. PLoS One. 2014; 25:e89908. 10.1371/journal.pone.0089908 PMC3934981 24587116

[31] International conference on harmonisation of technical requirements for registration of pharmaceuticals for human use, ICH harmonised tripartite guideline—Validation of Analytical Procedures: Text and Methodology—Q2 (R1), 2005.

[32] Donnelly R, Mooney K, Caffarel-Salvador E, Torrisi BM, Eltayib E, McElnay JC. Microneedle-mediated minimally invasive patient monitoring. Ther. Drug Monit. 2014; 36:10–17. 10.1097/FTD.0000000000000022 24365984

[33] Kiang TK, Schmitt V, Ensom MH, Chua B, Häfeli UO. Therapeutic drug monitoring in interstitial fluid: a feasibility study using a comprehensive panel of drugs. J Pharm Sci 2012; 101: 4642–4652. 10.1002/jps.23309 22941939

[34] Hafeli U, Ensom M, Kiang T, Stoeber B, Chua B, Pudek M et al. Comparison of Vancomycin concentrations in blood and interstitial fluid: A possible model for less invasive therapeutic drug monitoring. Clin Chem Lab Med 2011; 49:2123–2125. 10.1515/CCLM.2011.727 21942850

[35] Radomska-Botelho Moniz A, Michelakis K, Trzebinski J. Minimally invasive enzyme microprobes: an alternative approach for continuous glucose monitoring. J. Diabetes Sci. Technol. 2012; 6:479–480. 22538163 10.1177/193229681200600239 PMC3380796

[36] Trzebinski J, Sharma S, Moniz A, Michelakis K, Zhang Y. Microfluidic device to investigate factors affecting performance in biosensors designed for transdermal applications. Lab. Chip. 2012; 12:348–352. 10.1039/c1lc20885c 22130554

[37] Miller PR, Gittard SD, Edwards TL, Lopez DM, Xiao X, Wheeler DR et al. Multiplexed microneedle-based biosensor array for characterization of metabolic acidosis. Talanta. 2012; 88:739–742 10.1016/j.talanta.2011.11.046 22265568 PMC3266534

[38] Hamilton JG. Needle phobia: a neglected diagnosis. J. Fam. Pract. 1995; 41:169–175. 7636457

[39] Giudice EL, Campbell JD. Needle-free vaccine delivery. Adv. Drug Del. Rev. 2006; 58:68–89. 10.1016/j.addr.2005.12.003 16564111

[40] Kersten G, Hirschberg H. Needle-free vaccine delivery. Expert. Opin. Drug Deliv. 2007; 4:459–474. 17880271 10.1517/17425247.4.5.459

[41] Sekkat N, Naik A, Kalia Y N, Glikfeld P, Guy RH. Reverse iontophoretic monitoring in premature neonates: feasibility and potential. J. Contol. Release 2002; 81:83–89. 10.1016/s0168-3659(02)00046-9 11992681

[42] Bal S, Caussin J, Pavel S, Bouwstra J. *In vivo* assessment of safety of microneedle arrays in human skin. Eur. J. Pharm. Sci. 2008; 35:193–202. 10.1016/j.ejps.2008.06.016 18657610

[43] Prausnitz MR, Langer R. Transdermal drug delivery. Nat. Biotechnol. 2008; 26:1261–1268. 10.1038/nbt.1504 18997767 PMC2700785

[44] Nirogi RVS, Kandikere VN, Shukla M, Mudigonda K, Ajjala DR. A simple and rapid HPLC/UV method for the simultaneous quantification of theophylline and etofylline in human plasma. J. Chromatogr. B. 2007; 848:271–276. 10.1016/j.jchromb.2006.10.035 17110179

[45] Papadoyannis IN, Samanidou VF, Tsoukali H, Epivatianou F. Comparison of a RP-HPLC method with the therapeutic drug monitoring system TDx for the determination of theophylline in blood serum. Anal. Lett. 1993; 26:2127–2142.

[46] Evenson MA, Warren BL. Serum Theophylline analysis by high-pressure liquid chromatography. Clin Chem. 1976; 22:851–5. 1277472

[47] Habel D, Guermouche S, Guermouche MH. Direct determination of theophylline in human serum by high-performance liquid chromatography using zwitterionic micellar mobile phase. Comparison with an enzyme multiplied immunoassay technique. Analyst. 1993; 118:1511–3. 8109754 10.1039/an9931801511

[48] Lawson G, Patel P, Mulla H, Tanna S. Dried blood spot sampling with LC–MS analysis for routine therapeutic caffeine monitoring in neonates. ISRN Chromatography 2012; 828719, 7 pages.

[49] Umemura T, Kazumi-Inagaki K. Direct injection determination of theophylline and caffeine in blood serum by high-performance liquid chromatography using an ODS column coated with a zwitterionic bile acid derivative. Analyst. 1998; 123:1767–1770. 10071391 10.1039/a803153c

[50] Kenya Sakamoto FK. Usefulness of a novel system for measuring glucose area under the curve while screening for glucose intolerance in outpatients. J Diabetes Investig. 2013; 4:552–559. 10.1111/jdi.12096 24843709 PMC4020250

[51] Donnelly RF, McCrudden MT, Zaid-Alkilani A, Larrañeta E, McAlister E, Courtenay AJ et al. Hydrogel-forming microneedles prepared from "super swelling" polymers combined with lyophilised wafers for transdermal drug delivery. PLoS One. 2014; 9:e111547 10.1371/journal.pone.0111547 25360806 PMC4216095

[52] Garland MJ, Migalska K, Tuan-Mahmood TM, Thakur RRS, Majithija R, Caffarel-Salvador E et al. Influence of skin model on *in vitro* performance of drug-loaded soluble microneedle arrays. Int J Pharm. 2012; 434:80–9. 10.1016/j.ijpharm.2012.05.069 22669101

[53] Coffey JW, Corrie SR, Kendall MAF. Early circulating biomarker detection using a wearable microprojection array skin patch. Biomaterials. 2013; 34:9572–9583. 10.1016/j.biomaterials.2013.08.078 24044999

[54] Mahdavinia GR, Mousavi SB, Karimi F. Synthesis of porous poly(acrylamide) hydrogels using calcium carbonate and its application for slow release of potassium nitrate. Express Polym. Lett. 2009; 3:279–285.

[55] Peppas NA, Bures P, Leobandung W, Ichikawa H. Hydrogels in pharmaceutical formulations. Eur. J. Pharm. Biopharm. 2000; 50:27–46. 10840191 10.1016/s0939-6411(00)00090-4

[56] Romanyuk AV, Zvezdin VN, Samant P, Grenader MI, Zemlyanova M, Prausnitz MR. Collection of analytes from microneedle patches. Anal Chem. 2014; 86:10520–3. 10.1021/ac503823p 25367229 PMC4222632

[57] Ito Y, Taniguchi M, Hayashi A, Anai M, Morita S, Ko E et al. Application of dissolving microneedles to glucose monitoring through dermal interstitial fluid. Biol Pharm Bull. 2014; 37:1776–81. 25366483 10.1248/bpb.b14-00406

[58] Nwobodo N. Therapeutic drug monitoring in a developing nation: a clinical guide. JRSM Open. 2014; 8:5 10.1177/2054270414531121 PMC4100237 25289142

[59] Sakaguchi K, Hirota Y, Hashimoto N, Ogawa W, Sato T, Okada S et al. A minimally invasive system for glucose area under the curve measurement using interstitial fluid extraction technology: evaluation of the accuracy and usefulness with oral glucose tolerance tests in subjects with and without diabetes. Diabetes Technol. Ther. 2012; 14:485–491. 10.1089/dia.2011.0255 22537393

[60] Kume H, Hall IP, Washabau RJ, Takagi K, Kotlikoff MI. Beta-adrenergic agonists regulate KCa channels in airway smooth muscle by cAMP-dependent and -independent mechanisms. J Clin Invest. 1994; 93:371–9. 7904270 10.1172/JCI116969 PMC293787

[61] Ferapontova EE, Olsen EM, Gothelf KV. An RNA Aptamer-Based Electrochemical Biosensor for Detection of Theophylline in Serum. J. Am. Chem. Soc. 2008; 130:4256–4258. 10.1021/ja711326b 18324816

[62] Milavetz G, Vaughan L, Weinberger M, Hendeles L. Evaluation of a scheme for establishing and maintaining dosage of theophylline in ambulatory patients with chronic asthma. J. Pediatr. 1986; 109:351–354. 3734974 10.1016/s0022-3476(86)80403-6

[63] Piafsky KM, Ogilvie RI: Drug therapy. Dosage of theophylline in bronchial asthma. N. Engl. J. Med. 1975; 292:1218–1222. 1128573 10.1056/NEJM197506052922305

[64] Leung P, Kalisker A, Bell TD. Variation in theophylline clearance rate with time in chronic childhood asthma. J. Allergy Clin. Immunol. 1977; 59:440–444. 864102 10.1016/0091-6749(77)90007-0

[65] Yoshikawa H. First-line therapy for theophylline-associated seizures. Acta Neurol Scand. 2007; 115:57–61. 17362277 10.1111/j.1600-0404.2007.00810.x

[66] Teunissen MWE, Brorens ION, Geerlings JM, Breimer DD. Dose-dependent elimination of theophylline in rats. Xenobiotica. 1985; 15:165–171. 4002738 10.3109/00498258509045346

[67] Liguori A, Hughes JR, Grass JA. Absorption and subjective effects of caffeine from coffee, cola and capsules. Pharmacol Biochem Behav. 1997; 58:721–6. 9329065 10.1016/s0091-3057(97)00003-8

[68] Li CG, Lee CY, Lee K, Jung H. An optimized hollow microneedle for minimally invasive blood extraction. Biomed Microdevices. 2013; 15:17–25. 10.1007/s10544-012-9683-2 22833155

[69] Jina A, Tierney MJ, Tamada JA, McGill S, Desai S, Chua B et al. Design, development, and evaluation of a novel microneedle array-based continuous glucose monitor. J Diabetes Sci Technol. 2014; 8:483–487. 10.1177/1932296814526191 24876610 PMC4455438

[70] Wang PM, Cornwell M, Prausnitz MR. Minimally invasive extraction of dermal interstitial fluid for glucose monitoring using microneedles. Diabetes Technol Ther. 2005; 7:131–41. 15738711 10.1089/dia.2005.7.131

[71] Strambini LM, Longo A, Scarano S, Prescimone T, Palchetti I, Minunni M et al. Self-powered microneedle-based biosensors for pain-free high-accuracy measurement of glycaemia in interstitial fluid. Biosens Bioelectron. 2015; 66:162–8. 10.1016/j.bios.2014.11.010 25601169

[72] Groenendaal W, von Basum G, Schmidt KA, Hilbers PA, van Riel NA. Quantifying the composition of human skin for glucose sensor development. J Diabetes Sci Technol. 2010; 4:1032–40. 20920423 10.1177/193229681000400502 PMC2956818

[73] Basu A, Dube S, Slama M, Errazuriz I, Amezcua JC, Kudva YC et al. Time lag of glucose from intravascular to interstitial compartment in humans. Diabetes. 2013; 62:4083–7. 10.2337/db13-1132 24009261 PMC3837059

[74] Silber HE, Frey N, Karlsson MO. An integrated glucose-insulin model to describe oral glucose tolerance test data in healthy volunteers. J Clin Pharmacol. 2010; 50:246–56. 10.1177/0091270009341185 19940230

[75] Garg SK, Voelmle M, Gottlieb PA. Time lag characterization of two continuous glucose monitoring systems. Diabetes Res Clin Pract. 2010; 87:348–53. 10.1016/j.diabres.2009.11.014 20022127

[76] Yu J, Zhang Y, Ye Y, DiSanto R, Sun W, Ranson D et al. Microneedle-array patches loaded with hypoxia-sensitive vesicles provide fast glucose-responsive insulin delivery. Proc Natl Acad Sci USA. 2015; 112:8260–5. 10.1073/pnas.1505405112 26100900 PMC4500284

[77] Kiang TK, Schmitt V, Ensom MH, Chua B, Häfeli UO. Therapeutic drug monitoring in interstitial fluid: a feasibility study using a comprehensive panel of drugs. J Pharm Sci. 2012; 101:4642–52. 10.1002/jps.23309 22941939

